# Localized Outbreaks of Epidemic Polyarthritis among Military Personnel Caused by Different Sublineages of Ross River Virus, Northeastern Australia, 2016–2017

**DOI:** 10.3201/eid2510.181610

**Published:** 2019-10

**Authors:** Wenjun Liu, Joanne R. Kizu, Luke R. Le Grand, Christopher G. Moller, Tracy L. Carthew, Ian R. Mitchell, Ania J. Gubala, John G. Aaskov

**Affiliations:** Australian Defence Force Malaria and Infectious Disease Institute, Enoggera, Queensland, Australia (W. Liu, J.R. Kizu, L.R. Le Grand, C.G. Moller, T.L. Carthew);; Australian Defence Science and Technology Group, Fishermans Bend, Victoria, Australia (I.R. Mitchell, A.J. Gubala);; Queensland University of Technology, Brisbane, Queensland, Australia (J.G. Aaskov)

**Keywords:** Australian Defence Force, epidemic polyarthritis, outbreak, phylogenetic analysis, Ross River virus, Australia, viruses, military personnel, vector-borne infections, arboviruses

## Abstract

Two outbreaks of epidemic polyarthritis occurred among Australian Defence Force personnel during and following short military exercises in the Shoalwater Bay Training Area, northeastern Australia, in 2016 and 2017. Ross River virus (RRV) IgM was detected in acute-phase serum samples from most patients (28/28 in 2016 and 25/31 in 2017), and RRV was recovered from 4/38 serum samples assayed (1/21 in 2016 and 3/17 in 2017). Phylogenetic analyses of RRV envelope glycoprotein E2 and nonstructural protein nsP3 nucleotide sequences segregated the RRV isolates obtained in 2016 and 2017 outbreaks into 2 distinct sublineages, suggesting that each outbreak was caused by a different strain of RRV. The spatiotemporal characteristics of the 2016 outbreak suggested that some of the infections involved human-mosquito-human transmission without any intermediate host. These outbreaks highlight the importance of personal protective measures in preventing vectorborne diseases for which no vaccine or specific prophylaxis exists.

Epidemic polyarthritis (EPA) caused by Ross River virus (RRV) infection is the most frequently reported arboviral disease in Australia; ≈55,000 cases have been reported over the past decade (*1*). RRV is a positive-sense, single-strand RNA, enveloped virus in the *Alphavirus* genus of the Togaviridae family. Other viruses in this genus include chikungunya virus (CHIKV), Barmah Forest virus (BFV), Sindbis virus, and Eastern and Western equine encephalitis viruses. The prototype strain of RRV (T48) was isolated in 1959 from *Aedes vigilax* mosquitoes captured near the Ross River in Townsville, Queensland, Australia (*2*). Since then, outbreaks have been recorded in all the states of Australia (*3*) and in the South Pacific and Western Pacific regions, including Fiji (*4*), Samoa (*5*,*6*), the Cook Islands (*7*), New Caledonia (*8*), and Papua New Guinea (*9*,*10*). In Australia, most RRV infections occur during February–May or after periods of high rainfall or spring tides (*11*).

RRV is endemic and enzootic in Australia and has a natural animal-mosquito-animal transmission cycle. Humans can become infected incidentally by virus spillover, resulting in seasonal disease outbreaks. RRV has a complex ecology; >40 different mosquito species have been implicated as vectors, and >18 different wild and domestic animals and birds could serve as amplifying hosts (*12*–*14*). Humans can carry the virus from endemic to epizootic regions, and human-mosquito-human transmission is thought to be the most common means of transmission during large epidemics (*15*,*16*).

The RRV disease state varies widely between persons. This variation is readily apparent during outbreaks of the virus, wherein most persons in an infected population have asymptomatic or subclinical infection and only a few patients are substantially affected by the virus (ratio ≈3:1) (*4*). Among patients reporting clinical symptoms, most will recover in 4–6 weeks. However, in some cases, joint pain, muscle pain, and fatigue can persist for several months or years (*17*). RRV-caused EPA is characterized by arthritis, particularly in the small joints of the hands and feet. About 20%–60% of patients also have rash, fever, malaise, or a combination of these signs and symptoms (*18*–*21*). Symptoms similar to EPA occur after infection with BFV, CHIKV, Epstein-Barr virus (*22*), rubella virus (*23*), and parvovirus B19 (*24*). BFV co-circulates with RRV; ≈1,600 cases of BFV infection are reported each year in Australia (*1*,*20*). Although transmission of CHIKV does not occur in Australia, clinical infections have been reported in travelers returning to Australia from endemic areas (*25*,*26*).

During 2016–2017, two outbreaks of EPA occurred in Australian Defence Force (ADF) personnel during and after short military exercises in the Shoalwater Bay Training Area (SWBTA) in northeastern Australia. We conducted an investigation to confirm whether the 2 outbreaks of EPA among ADF personnel in SWBTA were attributable to RRV infection by identifying RRV RNA in patient serum samples and to determine whether the viruses were novel genotypes (and, if so, their phylogenetic origin).

## Material and Methods

### Ethics Statement

This study was a retrospective study approved by the ADF Joint Health Command Ethics Review Committee (Joint Health Command low-risk ethics panel no. 16-021). We obtained written formal consent from all participants.

### Study Area

SWBTA is a 4,545-km^2^ expanse of naturally vegetated coastal region ≈80 km north of the city of Rockhampton, Queensland, in northeastern Australia. ADF and allied forces use it regularly for military training. Because of its large size, restricted access, and protected ecology, regular mosquito surveillance or control is not conducted in the area. SWBTA is populated with large numbers of mammal and bird species, which could serve as hosts for RRV, as well as >40 species of mosquitoes, including major RRV vectors *Aedes vigilax* and *Culex annulirostris* mosquitoes (*11*,*18*,*27*). Weather conditions in SWBTA during March–May are typically hot and humid.

### Epidemiologic Data Collection

Laboratory confirmation of a RRV infection is achieved by isolating RRV or detecting viral RNA in patient serum samples or through observing seroconversion within 8–10 weeks of onset of symptoms consistent with RRV infection (*28*). Clinical records for patients could not be accessed for this investigation, so we collected information about clinical symptoms and what personal protective measures (PPMs) patients had undertaken during the exercise by using questionnaires completed by ADF personnel who had EPA symptoms and had given their consent to participate in this study.

### Virus Isolation and Genotyping

We obtained acute-phase serum samples from EPA patients who consented to participate in this study from Queensland Medical Laboratories on completion of routine RRV testing for RRV IgM and IgG with Panbio ELISA kits (http://www.panbiosystems.com). We recovered virus by culturing 100 μL of a patient serum sample on monolayers of C6–36 cells. We detected RRV infection in these cells 3 days postinfection by indirect immunofluorescence using an RRV-specific monoclonal antibody D7 (*29*).

We extracted viral RNA from serum and cell culture fluid by using the QIAamp Viral RNA Mini Kit (QIAGEN, https://www.qiagen.com) according to the manufacturer’s instructions. The glycoprotein E2 and nonstructural protein nsP3 genes were amplified by using reverse transcription PCR, and amplicons were purified from Tris-acetate-EDTA (TAE) agarose gels and sequenced at the Australian Genome Research Facility, as described previously (*30*). We edited and assembled all sequences by using Geneious 11.2 (https://www.geneious.com). An additional 20 strains of RRV collected previously by our laboratory were sequenced in the same manner and submitted to GenBank (Appendix Table 1).

### Phylogenetic Analysis

We aligned nucleotide sequences of 58 RRV E2 genes and 32 nsP3 genes (Appendix Table 1) by using the ClustalW program in Geneious. E2 and nsP3 sequences for 24 of these viruses were derived in this study. We converted the alignment file to NEXUS format by using MEGAX for use with BEAST (http://www.beast.community) and associated tools to postulate a phylogenetic tree for the RRV E2 and nsP3 proteins. We determined the nucleotide-substitution model by using the model test capability in MEGAX and confirmed the findings by using jModelTest 2.1.3 (*31*). Although the general time reversible plus gamma distribution with invariant sites (GTR+Γ+I) and Hasegawa–Kishono–Yano (HKY) substitution models were considered in BEAST, TN93+Γ had the lowest Bayesian information criterion score (*32*–*34*) (Appendix Table 2) and was selected for the phylogenetic analysis of the E2 gene.

Isolation dates of RRV at the tips of phylogenetic trees were taken from the GenBank “collection date” field and estimated to have a precision of +1.5 years. We conducted initial analyses assuming a strict molecular clock model because the evolutionary timeframe for this study was comparatively short. However, we also acknowledged that the environmental conditions were sufficiently diverse to warrant a relaxed clock (lognormal). We used the Bayesian skyline as the demographic model for the phylogenetic trees. We performed Markov chain Monte Carlo analysis by using BEAST version 1.8.1 with a 10 million chain length and sampling every 1,000 generations and assessed convergence of parameters on the basis of the ESS value >200, which was viewed by using Tracer 1.6.0. We subsequently generated maximum clade credibility trees after a 10% burn-in protocol by using TreeAnnotator version 1.8.1 and formatted the final trees in FigTree 1.3.1. We obtained all software for these analyses, except Geneious, from http://www.beast.community.

## Results

**Figure 1:**
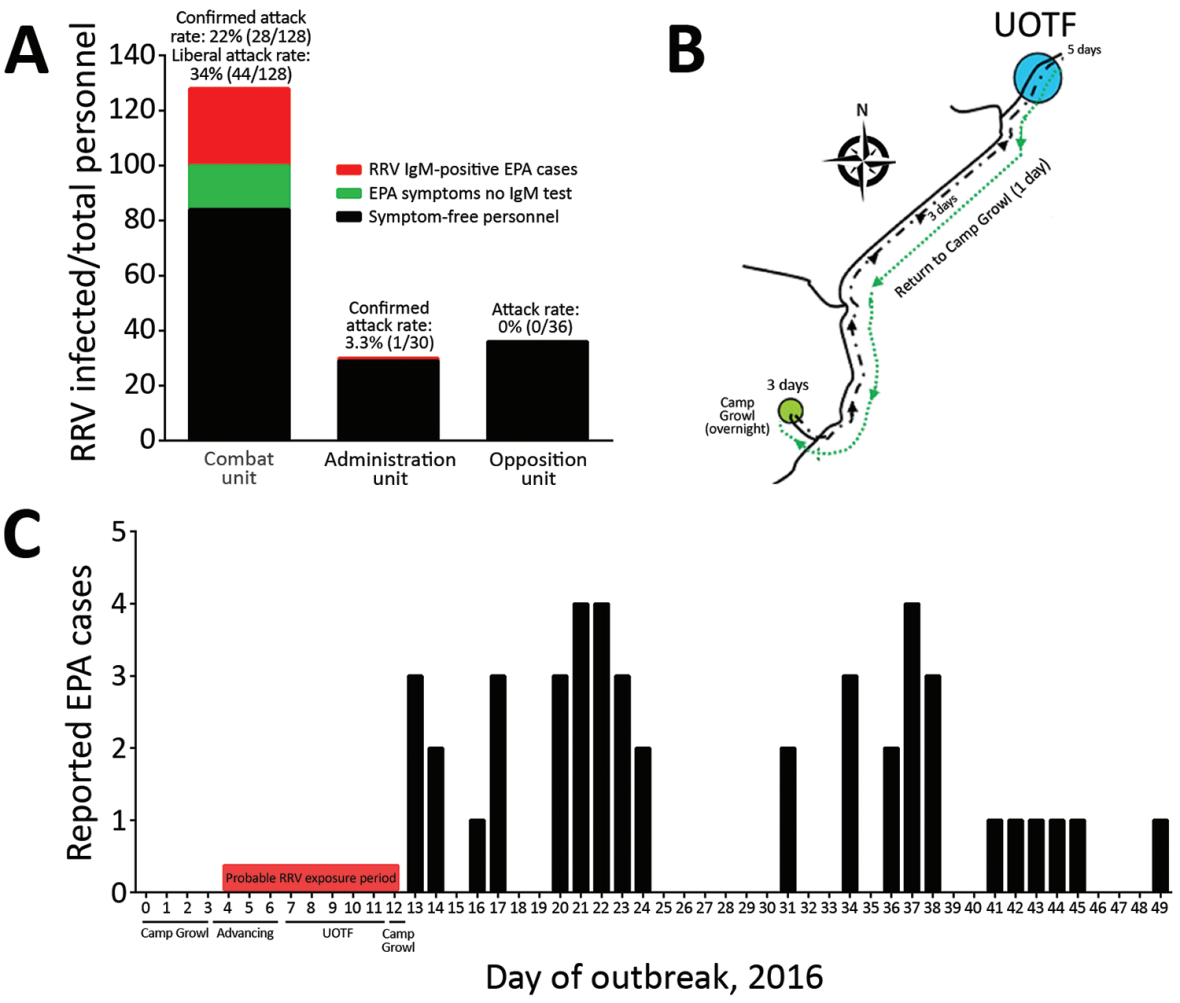
Characteristics of an RRV outbreak among Australian Defence Force (ADF) personnel during and after training in Shoalwater Bay Training Area (SWBTA), northeastern Australia, 2016. A) RRV attack rates among 3 ADF units. B) Routes of ADF units during exercises in SWBTA. C) Timeline of outbreak. EPA, epidemic polyarthritis; RRV, Ross River virus; UOTF, urban operation training facility.

The first EPA outbreak was reported after a 12-day exercise (February 29–March 11, 2016). Forty-four personnel from a combat unit of 128 sought care at the Regimental Aid Post (RAP) with rash, headache, nausea, fatigue, lethargy, and joint and muscle pain at the conclusion of the exercise. RRV IgM was detected in acute- or convalescent-phase serum samples collected from 28 of these 44 persons 14–50 days after symptom onset (Table); attack rates ranged from 22% (confirmed cases only, 28/128 [22%]) to 34% (all suspected cases, 44/128 [34%]) (Figure 1, panel A).

**Table:** Epidemiologic characteristics of RRV outbreaks among ADF personnel during and after training in SWBTA, northeastern Australia, 2016–2017*

Characteristic	Outbreak	
	2016	2017
Total no. reported EPA cases	44	43
% RRV IgM antibody–reactive cases/tested cases	100 (28/28)	80.6 (25/31)
No. participants who consented	21	17
Average age, y (range)	ND	27 (20–45)
Sex, no.	ND	16 M, 1 F
History of arbovirus infection before this infection	0	0
Outdoor training experience in SWBTA	100 (21)	100 (17)
Clinical symptoms		
Severe headache	28.6 (6/21)	41.2 (7/17)
Nausea	4.8 (1/21)	17.7 (3/17)
Rash	9.5 (2/21)	64.7 (11/17)
Fever, chills, or sweats	33.3 (7/21)	47.1 (8/17)
Arthralgia	66.7 (14/21)	88.2 (15/17)
Muscle pain	71.4 (15/21)	88.2 (15/17)
Fatigue	71.4 (15/21)	88.2 (15/17)
Loss of appetite	52.4 (11/21)	70.1 (12/17)
Stiff neck	38.1 (8/21)	64.7 (11/17)
Mosquito bite experiences	100 (21/21)	100 (17/17)
Personal protection measures		
Mosquito repellents	100 (21/21)	100 (17/17)
Trousers and long-sleeve shirts	85.7 (18/21)	47.1 (8/17)
Slept under ADF-issued mosquito nets	57.1 (12/21)	82.4 (14/17)
Awareness of permethrin uniform and bed net treatment	100 (21/21)	100 (17/17)
Use of ADF-recommended permethrin treatment	0 (0/21)	0 (0/17)

*Data are % (no. positive/total no.) unless otherwise indicated. ADF, Australian Defence Force; EPA, epidemic polyarthritis; ND, not done; RRV, Ross River virus; SWBTA, Shoalwater Bay Training Area.

Troops from the affected unit (referred to hereafter as the combat unit) had spent 3 days in heavy rain near Camp Growl in the training area (Figure 1, panel B). During the following 3 days, the unit moved over varied terrain, including dense bushland, in hot and humid conditions to an urban operation training facility (UOTF). Once at the UOTF, they conducted urban assault maneuvers against a 36-member opposition force for an additional 5 days. Upon conclusion of the UOTF component of the exercise, the combat unit marched back to Camp Growl, where they spent the night before returning to Brisbane, ≈630 km south of SWBTA. An administration unit of 30 persons was stationed in SWBTA during the same period but remained at Camp Growl throughout the exercise. In addition to the 44 members of the combat unit affected by EPA, 1 member from the administration unit reported symptoms of EPA, and RRV IgM was detected in this patient’s serum sample upon return to Brisbane (attack rate 3.3%) (Figure 1, panel A). None of the 36 members of the opposition force reported symptoms of EPA (Figure 1, panel A). This localized EPA outbreak had 2 distinct epidemic curves, with gaps of 7 days and ≈15 days between the peaks (Figure 1, panel C).

In 2017, a total of 43 members from 3 different units had symptoms of EPA during an exercise conducted from late April to mid-May in the same area as the combat unit affected in the 2016 outbreak. Thirty-one of the 43 patients provided a serum sample. Of these, 25 samples contained RRV IgM (Table). Neither the advancing routes of these 3 units through SWBTA nor the epidemiologic timeline of the 2017 EPA outbreak could be obtained because of ADF operational restrictions. Additional EPA cases might have occurred during both outbreaks, given that anecdotal evidence suggests that members who were unwell at that time chose not to seek care at the RAP. ADF members were not screened for RRV seroconversions over the course of the exercise, so asymptomatic RRV infections could not be detected. Because most RRV infections are asymptomatic, the number of infections during these outbreaks was probably higher than the number of cases reported.

The most common symptoms experienced in the 2016 and 2017 outbreaks were, respectively, polyarthritis and muscle pain (71% and 88%), arthralgia (67% and 88%), fatigue (71% and 88%), loss of appetite (52% and 75%), stiff neck (38% and 71%), fever (33% and 47%), rash (9.5% and 65%), headache (28% and 41%), and sore throat (14% and 24%) (Table). Most personnel recovered within 4–6 weeks of symptom onset, except for 3 from the 2016 outbreak who were unfit for deployment 3 months after illness onset because of ongoing signs and symptoms.

All study participants were aware of the ADF policy of dipping uniforms and bed nets in permethrin, but none of the participants, nor their unit commanders, requested support from the Preventive Medicine Company to undertake this procedure before or during either exercise, citing time constraints (Table). More than half of the personnel failed to comply with the sleeves-down policy because of the hot and humid weather conditions. All participants stated that they used commercial repellents, which contain relatively low concentrations of N,N-Diethyl-meta-toluamide (DEET) compared with the repellents issued by the ADF. About 57% (2016) and 82% (2017) of personnel reported sleeping under bed nets at night but noted that nets at times were abandoned because of their incompatibility with the tactical situation. Five soldiers from the 2017 outbreak reported constantly being assailed with mosquito bites (concentrated around their hands and legs) during the night even when inside their nets. Gloves and socks were worn when sleeping to address this issue. All participants reported being bitten by mosquitoes on a regular basis.

**Figure 2:**
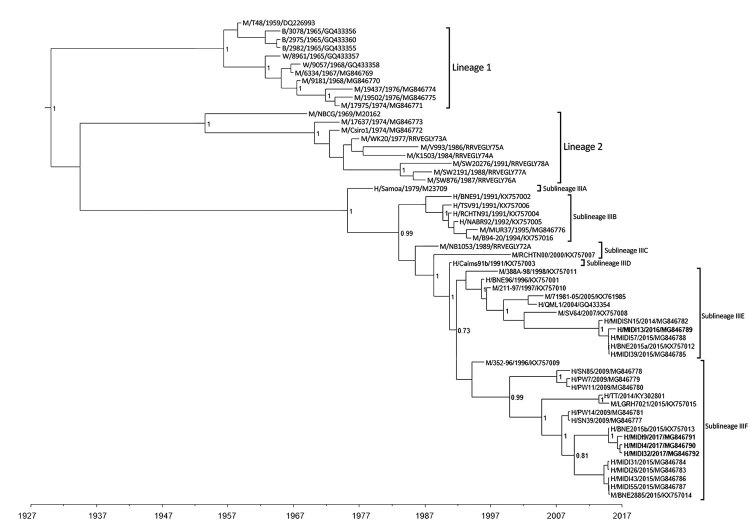
Maximum clade credibility tree based on analysis of 58 complete Ross River virus E2 sequences (1,266 nt) from outbreaks among Australian Defence Force personnel during and after training in Shoalwater Bay Training Area, northeastern Australia, 2016–2017. Isolates were classified into 2 distinct sublineages in lineage III. We used Bayesian phylogenetic analysis method in BEAST software (http://beast.community) to analyze the aligned E2 sequences, applying the TN93 plus gamma substitution model with a strict clock model, a chain length of 10 million, and a 10% burn-in using TreeAnnotator (https://beast.community/treeannotator). Numbers at nodes indicate the posterior probability values >0.8 except the value for the bifurcation of the sublineages IIIE and IIIF. The naming convention of the strains was name of host/strain/year of isolation/GenBank accession number. B, birds; H, humans; M, mosquitoes; W, wallabies.

Bayesian phylogenetic analyses of 58 RRV complete E2 sequences (1,266 nt) were performed by using TN93+Γ, HKY+Γ, and the GTR+Γ+I substitution models with both strict and relaxed clock models. All phylogenies placed the ADF RRV isolates collected in 2016 and 2017 (MIDI13.2016, MIDI4.2017, MIDI9.2017, and MIDI32.2017) into 2 distinct sublineages of lineage III (IIIE and IIIF). The TN93+Γ substitution model was the most highly ranked of those used, having the lowest Bayesian information criterion score (*32*–*34*) (Appendix Table 1), and coupled with the strict clock to produce the E2 phylogenetic tree (Figure 2). The remaining trees are provided in Appendix Figures 1–5. 

**Figure 3:**
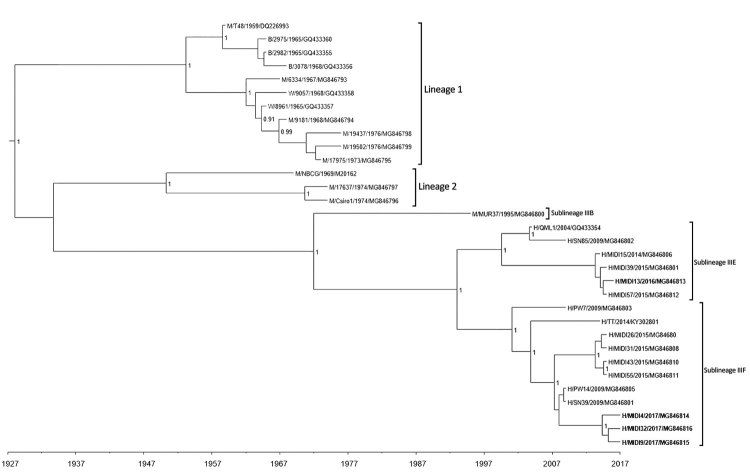
Maximum clade credibility tree based on analysis of 32 complete Ross River virus nsP3 sequences (1,650 nt) from outbreaks among Australian Defence Force personnel during and after training in Shoalwater Bay Training Area, northeastern Australia, 2016–2017. Isolates were classified into 2 distinct sublineages in lineage III. We used Bayesian phylogenetic analysis method in BEAST software (http://beast.community) to analyze the aligned nsP3 sequences, applying the TN93 plus gamma substitution model with a strict clock model, a chain length of 10 million, and a 10% burn-in using TreeAnnotator (https://beast.community/treeannotator). Numbers at nodes indicate the posterior probability values >0.8. The naming convention of the strains was name of host/strain/year of isolation/GenBank accession number. B, birds; H, humans; M, mosquitoes; W, wallabies.

Although the confidence level for the separation of the 2 sublineages differs depending on the model used (posterior probabilities range from 0.63 for HKY+Γ relaxed clock to 0.89 for GTR+Γ+I relaxed clock), the bifurcation of the 2 sublineages occurs in all of the trees produced (Figure 2; Appendix Figures 1–5). Furthermore, an analysis of 32 nsP3 sequences (1,650 nt) similarly demonstrates the bifurcation of the 2 proposed sublineages but with high posterior probability (≈1) for each of the HKY and GTR+Γ+I substitutional models with both strict and relaxed clocks. The tree for nsP3 based on the HKY substitution model with a strict clock (Figure 3) is also presented with alternative substitution models and clocks (Appendix Figures 6–8). 

Collectively, these results suggest that different lineages of RRV were responsible for the 2016 and 2017 outbreaks. The 2016 isolate (MIDI13.2016) resembled strains of RRV recovered from patients in Brisbane (≈600 km south of SWBTA) in 2014 and 2015 and belongs to sublineage IIIE. Lineage III is thought to have evolved from 2 other lineages of RRV that are believed to be extinct. The 3 isolates recovered in 2017 (MIDI4.2017, MIDI9.2017, and MIDI32.2017) shared 99.8% nucleotide identity in pairwise comparisons and have been assigned to sublineage IIIF, which contains strains of RRV recovered from the east and west coasts of Australia in 2009 and in an isolate identified in Brisbane in 2015 (BNE 2885.2015).

We observed 6 amino acid differences between the 2016 (sublineage IIIE) and 2017 (sublineage IIIF) isolates from SWBTA (N132D, Y296H, T369A, I376M, A384T, and A389T) of which only 1 (I376M) was a conservative change. All substitutions are located in the A and C domains of the E2 protein, in areas involved in the interaction with other proteins (E1, capsid, and 6k), as well as in the process of budding of alphavirus envelope proteins from host cell membranes (*35*,*36*). The 2 glycosylation sites on the E2 protein were conserved in all 4 of the SWBTA isolates.

## Discussion

The state of Queensland, where SWBTA is situated, has the highest rates of EPA in Australia, consistently recording >1,000 cases each year (*1*). RRV-caused EPA cases are reported routinely in the areas surrounding Rockhampton (*37*), the nearest city to SWBTA. In 1997, nineteen RRV-caused EPA cases were reported among US Navy personnel during a joint Australia–US military exercise located at SWBTA (*27*). In 2004, RRV RNA was detected in multiple mosquito species collected in this training area (*27*,*38*). The most recent common ancestor of RRV (strain T48) originated from northeastern Australia (*2*,*18*), and this finding is in agreement with our phylogenetic analysis (Figure 2). 

In light of these data, we reasonably believe that RRV has established its natural endemic cycle in SWBTA. The recurrent outbreaks of RRV infection within SWBTA and the intermittent presence of humans in this region suggests that the 2016 and 2017 infections were spillover events in which the virus was incidentally acquired by ADF soldiers from the natural endemic animal-mosquito-animal transmission cycle made possible by large populations of native animals (e.g., kangaroos and wallabies) and the wide variety of RRV vectors (*21*). However, phylogenetic analyses of nucleotide sequences of the E2 and nsP3 genes of RRV recovered from the ADF personnel in our study and from patients and mosquitoes from other parts of Australia do not support the notion of RRV strains being sequestered in endemic pockets across Australia. Instead, SWBTA isolates bore a remarkable similarity to those circulating in the wider community (Figures 2, 3; Appendix Figures 1–8). A more likely explanation is that strains of RRV are not regionally locked but spread around Australia by the movement of humans, as most likely occurred in the RRV outbreak in the Pacific during 1979–1980 (*4**–**8*). More surveillance and exhaustive genetic analysis might help to resolve the precise origin and nature of these outbreaks.

The spatiotemporal characteristics of the 2016 outbreak suggested that the virus was transmitted among soldiers by local mosquitoes after the initial infection. The first wave of soldiers who sought care at the RAP at Gallipoli Barracks, Brisbane, did so within a 2-week period of leaving the SWBTA, which is consistent with the 2–15-day incubation period proposed for RRV infection in humans (*18*) (Figure 1, panel C). Although this first wave of soldiers might have been infected with RRV by spillover from the suspected natural, endemic animal–mosquito–animal transmission cycle in SWBTA, few (if any) intermediate host-macropods exist in Gallipoli Barracks from which soldiers would become infected upon their return. Soldiers who sought care for EPA signs and symptoms 19–37 days after returning to the barracks are indicative of a potential secondary human-mosquito-human infection cycle in Gallipoli Barracks, possibly involving asymptomatic but viremic persons. This conclusion is further supported by the high level of homology shared by 3 RRV isolates from 2017. 

No EPA cases were reported (except 1 case from the administration unit attached to the combat team at SWBTA) from other units at Gallipoli Barracks (a base containing >5,600 personnel who operate across 30 different units) upon the return of the infected personnel. This finding might simply reflect the probability of an extremely small number of infected mosquitoes finding a susceptible and unprotected human host among other units headquartered at discrete localities spread over an area of >200 hectares. However, without concurrent isolation of the virus from local mosquitoes in the vicinity of Gallipoli Barracks, this 2-wave theory, however plausible, remains speculative. Delayed or failed reporting of health concerns or symptoms of infected personnel can be a confounding issue when surveying soldiers attached to combat units. This difficulty is caused in part by a cultural resistance to seek medical attention for ailments of low to moderate severity; soldiers often opt to press on in the face of adversity.

A previous study based on phylogenies derived from short E2 gene sequences identified 2 lineages of RRV (*3*), whereas a more recent study (*39*) using longer sequences identified 3 (1 from eastern Australia, 1 from western Australia, and 1 from northeastern Australia). The phylogenetic analyses in our study, which used the complete nucleotide sequences of RRV E2 and nsP3 genes, confirm the presence of 3 lineages but suggest that lineage III should be divided into 6 sublineages. Lineage I viruses were classified as the northeastern Australia lineage; examples of this lineage have not been isolated since 1977. Lineage II was classified as the western Australia lineage; examples of this lineage have not been isolated since 1991 (Figures 2, 3; Appendix Table 1, Figures 1–8). The virus strain of RRV that was responsible for the outbreaks in Fiji, the Cook Islands, and Samoa during 1979–1980 has been classified as sublineage IIIA. The results of our study suggest that the lineage III viruses have been responsible for outbreaks of RRV infection in Australia since 1991. The temporal structure of the maximum clade credibility trees suggest that RRV is continuing to diversify under the pressure of natural selection and potential for further diversification exists given changes in the virus’ ecologic niche, cycles of transmission, or both. The importance of the 5 nonconservative changes in the amino acid sequences of the E2 proteins that occurred from 2016 to 2017 warrants further investigation to determine what role they might play in viral fitness. The implementation of continuous and routine viral sequencing, analysis, and monitoring will increase confidence in the proposed delineation of the sublineages within lineage III and further clarify the evolutionary process.

With the ADF’s commitment to maintaining the natural environment in its training areas, an ongoing risk for infection with RRV persists for personnel who do not have natural immunity through prior exposure to RRV and who are required to spend prolonged periods in this RRV-endemic area. A further risk is the potential for RRV to be exported to other countries through infected, asymptomatic personnel participating in multinational exercises, which occur on a regular basis in SWBTA. This risk is of particular concern for countries with mosquitoes known to be RRV vectors (*40*). The largest outbreak of RRV infection ever recorded occurred in the Pacific during 1979–1980 (*4*) and is a testament to the epidemic potential of the virus. The recent experience with Zika and chikungunya viruses underscores the serious threat posed to global health by the potential for previously obscure arboviruses to move from their historical cycles of transmission (*41*,*42*).

In conclusion, this investigation confirmed through viral isolation and sequence analysis that the outbreaks of EPA in the ADF in 2016 and 2017 were caused by 2 distinct sublineages of lineage III strains of RRV. Further, these outbreaks are most likely attributable to human-mosquito-human transmission.

Appendix Additional information regarding localized outbreaks of epidemic polyarthritis among military personnel caused by different sublineages of Ross River virus, Northeast Australia, 2016–2017.
