# Supplementary material for: Localized Outbreaks of Epidemic Polyarthritis among Military Personnel Caused by Different Sublineages of Ross River Virus, Northeastern Australia, 2016–2017
Source: Emerg Infect Dis. 2019 Oct;25(10):1793–801. doi: 10.3201/eid2510.181610 (PMC6759257; doi:10.3201/eid2510.181610)
Supplement: Appendix — Additional information regarding localized outbreaks of epidemic polyarthritis among military personnel caused by different sublineages of Ross River virus, Northeast Australia, 2016–2017. [file 18-1610-Techapp-s1.pdf]

Article DOI: https://doi.org/10.3201/eid2510.181610

Localized Outbreaks of Epidemic Polyarthritis among Military Personnel Caused by Different Sublineages of Ross River Virus, Northeastern Australia, 2016–2017

Appendix

Appendix Table 1. Details of RRV strains used for phylogenetic study

No.	Isolate	Location of isolation	Host	Year isolated	E2 GenBank no.	nsP3 GenBank no.	Lineage
1	T48	Townsville, QLD, AUS	*Ae. vigilax*	1959	DQ226993	DQ226993	I
2	2982	Northeast, AUS	*Microeca fascinans*	1965	GQ433355	GQ433355	I
3	3078	Northeast, AUS	Poephila personata	1965	GQ433356	GQ433356	I
4	8961	Northeast, AUS	Macropus agilis	1965	GQ433357	GQ433357	I
5	2975	northeast, AUS	*Grallina cyanoleuca*	1965	GQ433360	GQ433360	I
6	6334	Koyanyama,QLD, AUS	*Culex annulirostris*	1967	MG846769	MG846793	I
7	9181	Koyanyama,QLD, AUS	*Culex annulirostris*	1968	MG846770	MG846794	I
8	9057	Northeast, AUS	*Macropus agilis*	1968	GQ433358	GQ433358	I
9	17975	Koyanyama,QLD, AUS	*Anopheles amitus*	1974	MG846771	MG846795	I
10	19437	Charleville, QLD, AUS	*Ae. normanensis*	1976	MG846774	MG846798	I
11	19502	Charleville, QLD, AUS	*Ae. normanensis*	1976	MG846775	MG846799	I
12	NBCG	Nelson Bay, NSW	*Mosquito*	1969	M20162	M20162	II
13	17637	Koyanyama,QLD, AUS	*Culex annulirostris*	1974	MG846773	MG846797	II
14	CSIRO	QLD, AUS	*Ae. normanensis*	1974	MG846772	MG846796	II
15	WK20	Northwest, WA, AUS	*Culex annulirostris*	1977	RRVEGLY73A	NA	II
16	K1503	Northwest, WA, AUS	*Culex annulirostris*	1984	RRVEGLY74A	NA	II
17	V993	North Territory, AUS	*Ae. vigilax*	1986	RRVEGLY75A	NA	II
18	SW876	Southwest, WA, AUS	*Ae. camptorhynchus*	1987	RRVEGLY76A	NA	II
19	SW2191	Southwest, WA, AUS	*Ae. camptorhynchus*	1988	RRVEGLY77A	NA	II
20	SW20276	Southwest, WA, AUS	*Ae. camptorhynchus*	1991	RRVEGLY78A	NA	II
21	Samoa	Samoa	Human	1979	M23709	NA	III
22	NB1053	Central Coast, NSW, AUS	Mixed *Ae.* and *Cx.* sp.	1989	RRVEGLY72A	NA	III
23	RCHTN91	Rockhampton, QLD, AUS	Human	1991	KX757004	NA	III
24	TSV91	Townsville, QLD, AUS	Human	1991	KX757006	NA	III
25	BNE91	Brisbane, QLD, AUS	Human	1991	KX757002	NA	III
26	Cairns91b	Cairns, QLD, AUS	Human	1991	KX757003	NA	III
27	NABR92	Nambour, QLD, AUS	Human	1992	KX757005	NA	III
28	B94/20	Brisbane, QLD, AUS	Mosquito	1994	KX757016	NA	III
29	37MUR	Murwillumbah, NSW, AUS	*Ae. normanensis*	1995	MG846776	MG846800	III
30	BNE96	Brisbane, QLD, AUS	Human	1996	KX757001	NA	III
31	352/96	Cairns, QLD, AUS	Mosquito	1996	KX757009	NA	III
32	211-97	Cairns, QLD, AUS	Mosquito	1997	KX757010	NA	III
33	388A-98	Cairns, QLD, AUS	Mosquito	1998	KX757011	NA	III
34	RCHTN00	Rockhampton, QLD, AUS	Mosquito	2000	KX757007	NA	III
35	QML1	Northeast, QLD, AUS	Human	2004	GQ433354	GQ433354	III
36	71981-05	Port Stephens, NSW, AUS	Mosquito	2005	KX761985	NA	III
37	SV64	Cairns, QLD, AUS	Mosquito	2007	KX757008	NA	III
38	SN39	Yamba, NSW, AUS	Human	2009	MG846777	MG846801	III
39	SN85	Middlemount, QLD, AUS	Human	2009	MG846778	MG846802	III
40	PW7	Esperance, WA, AUS	Human	2009	MG846779	MG846803	III
41	PW11	Broome, WA, AUS	Human	2009	MG846780	NA	III
42	PW14	Karratha,WA, AUS	Human	2009	MG846781	MG846805	III
43	LGRH7021	Longreach, QLD, AUS	Mosquito	2013	KX757015	NA	III
44	MIDISN15.2014	Brisbane, QLD, AUS	Human	2014	MG846782	MG846806	III

No.	Isolate	Location of isolation	Host	Year isolated	E2 GenBank no.	nsP3 GenBank no.	Lineage
45	RRV_TT	Brisbane, QLD, AUS	Human (patient)	2014	KY302801	KY302801	III
46	MIDI39.2015	Brisbane, QLD, AUS	Human	2015	MG846785	MG846809	III
47	MIDI57.2015	Brisbane, QLD, AUS	Human	2015	MG846788	MG846812	III
48	BNE2015a	Brisbane, QLD, AUS	Human	2015	KX757012	NA	III
49	MIDI26.2015	Brisbane, QLD, AUS	Human	2015	MG846783	MG846807	III
50	MIDI31.2015	Brisbane, QLD, AUS	Human	2015	MG846784	MG846808	III
51	MIDI43.2015	Brisbane, QLD, AUS	Human	2015	MG846786	MG846810	III
52	MIDI55.2015	Brisbane, QLD, AUS	Human	2015	MG846787	MG846811	III
53	BNE2885	Brisbane, QLD,AUS	Mosquito	2015	KX757014	NA	III
54	BNE2015b	Brisbane, QLD, AUS	Human (serum)	2015	KX757013	NA	III
55	MIDI13.2016	Shoalwater Bay, QLD, AUS	Human	2016	MG846789	MG846813	III
56	MIDI4.2017	Shoalwater Bay, QLD, AUS	Human	2017	MG846790	MG846814	III
57	MIDI9.2017	Shoalwater Bay, QLD, AUS	Human	2017	MG846791	MG846815	III
58	MIDI32.2017	Shoalwater Bay, QLD, AUS	Human	2017	MG846792	MG846816	III

*NA, not available; NSW, New South Wales; QLD, Queensland; WA, Western Australia.

Appendix Table 2. Posterior probabilities for the bifurcation of the IIIE and IIIF sub-lineages for the proteins and models presented*

			Posterior Probability	
Protein	Substitution Models	BIC	Strict Clock	Relaxed Clock
E2	TN93+G	7371.5	0.73	0.67
	HKY+G	7377.1	0.71	0.63
	GTR+G+I	7407.1	0.8	0.89
nsP3	HKY	2452.2	1.0	1.0
	GTR+G+I	2502.0	1.0	1.0

*Lower BIC (Bayesian Information Criterion) scores indicate the models that better represent the substitution process. Posterior probabilities of 0.8 or greater are considered strong evidence for the bifurcation of a clade. Low posterior probability values are bolded, indicating lower confidence in the separation of the clades.

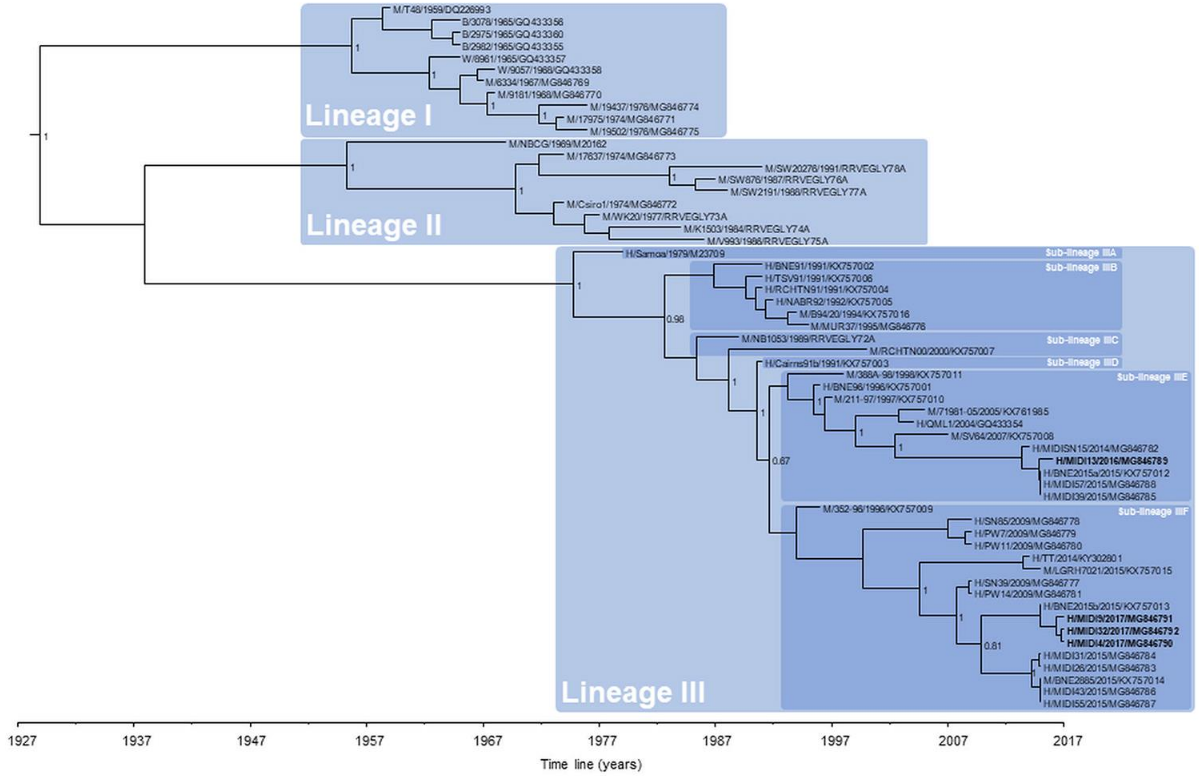

Appendix Figure 1. Maximum clade credibility tree based on analysis of 58 complete RRV E2 sequences (1266nt) classified ADF SWBTA RRV isolates (highlighted) into two distinct sub-lineages in lineage III. Bayesian phylogenetic analysis method in BEAST software package (http://beast.community)

was used to analysis the aligned E2 sequences, employ the TN93+G substitution model with a relaxed clock model. Numbers at nodes indicate the posterior probability values ≥0.8 except the value for the bifurcation of IIIE and IIIF sub-lineages. The x axis represents time in years. The naming convention of the strains were name of host/strain/year of isolation/GenBank accession number, H-human, B-birds, M-mosquitoes and W-wallabies.

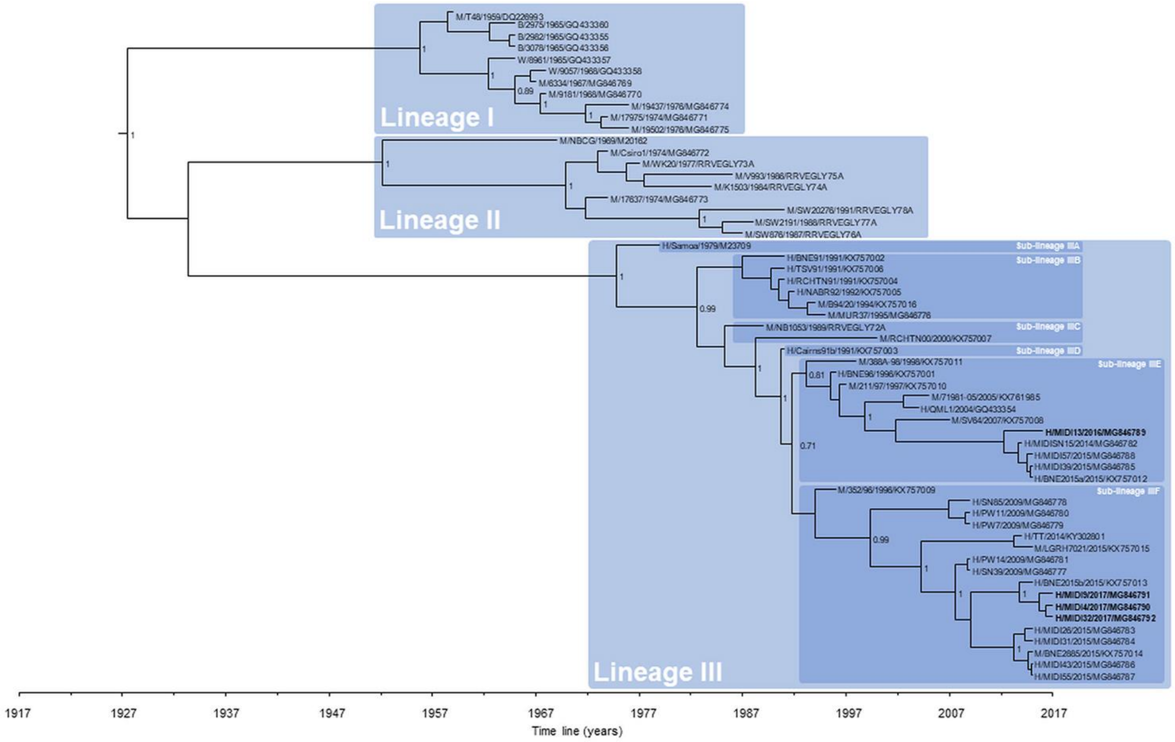

Appendix Figure 2. Maximum clade credibility tree based on analysis of 58 complete RRV E2 sequences (1266nt) classified ADF SWBTA RRV isolates (highlighted) into two distinct sub-lineages in lineage III. Bayesian phylogenetic analysis method in BEAST software package (http://beast.community) was used to analysis the aligned E2 sequences, employ the HKY+G substitution model with a strict clock model. Numbers at nodes indicate the posterior probability values ≥0.8 except the value for the bifurcation of IIIE and IIIF sub-lineages. The x axis represents time in years. The naming convention of the strains were name of host/strain/year of isolation/GenBank accession number, H-human, B-birds, M-mosquitoes and W-wallabies.

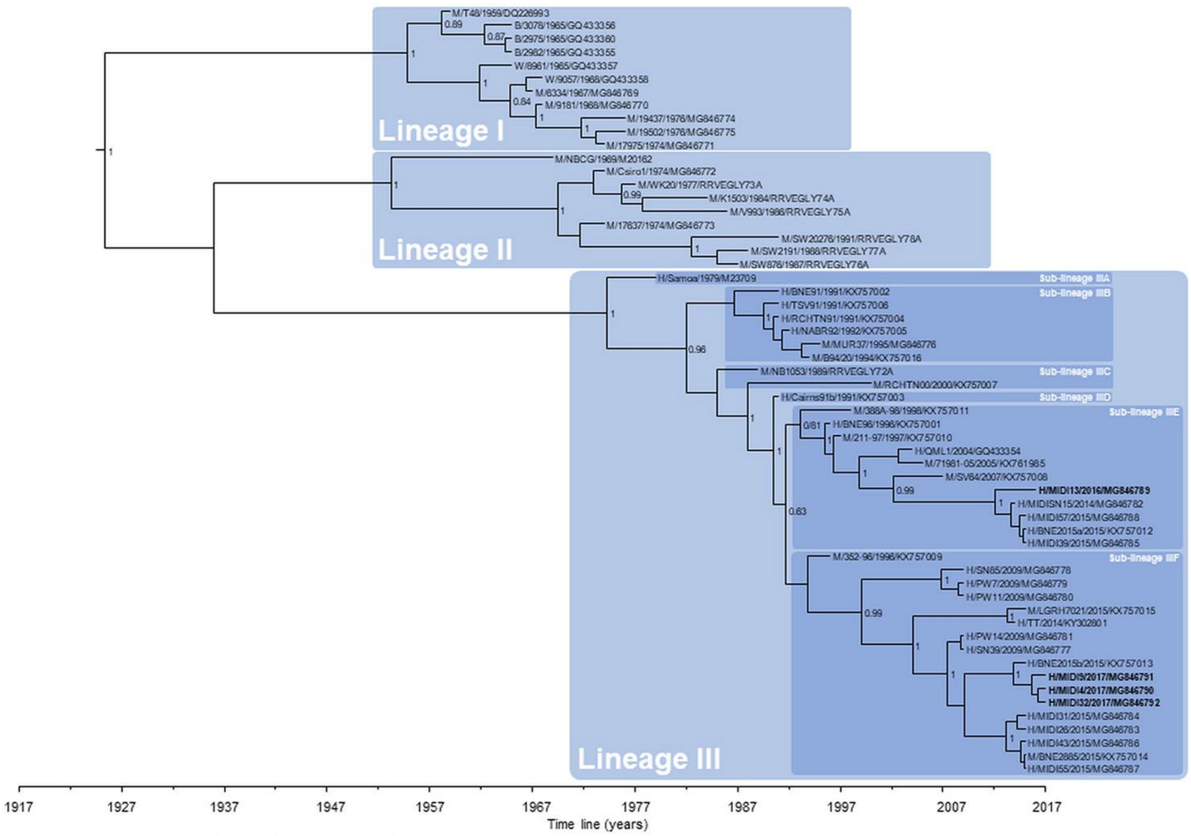

Appendix Figure 3. Maximum clade credibility tree based on analysis of 58 complete RRV E2 sequences (1266nt) classified ADF SWBTA RRV isolates (highlighted) into two distinct sub-lineages in lineage III. Bayesian phylogenetic analysis method in BEAST software package (http://beast.community) was used to analysis the aligned E2 sequences, employ the HKY+G substitution model with a relaxed clock model. Numbers at nodes indicate the posterior probability values ≥0.8 except the value for the bifurcation of IIIE and IIIF sub-lineages. The x axis represents time in years. The naming convention of the strains were name of host/strain/year of isolation/GenBank accession number, H-human, B-birds, M-mosquitoes and W-wallabies.

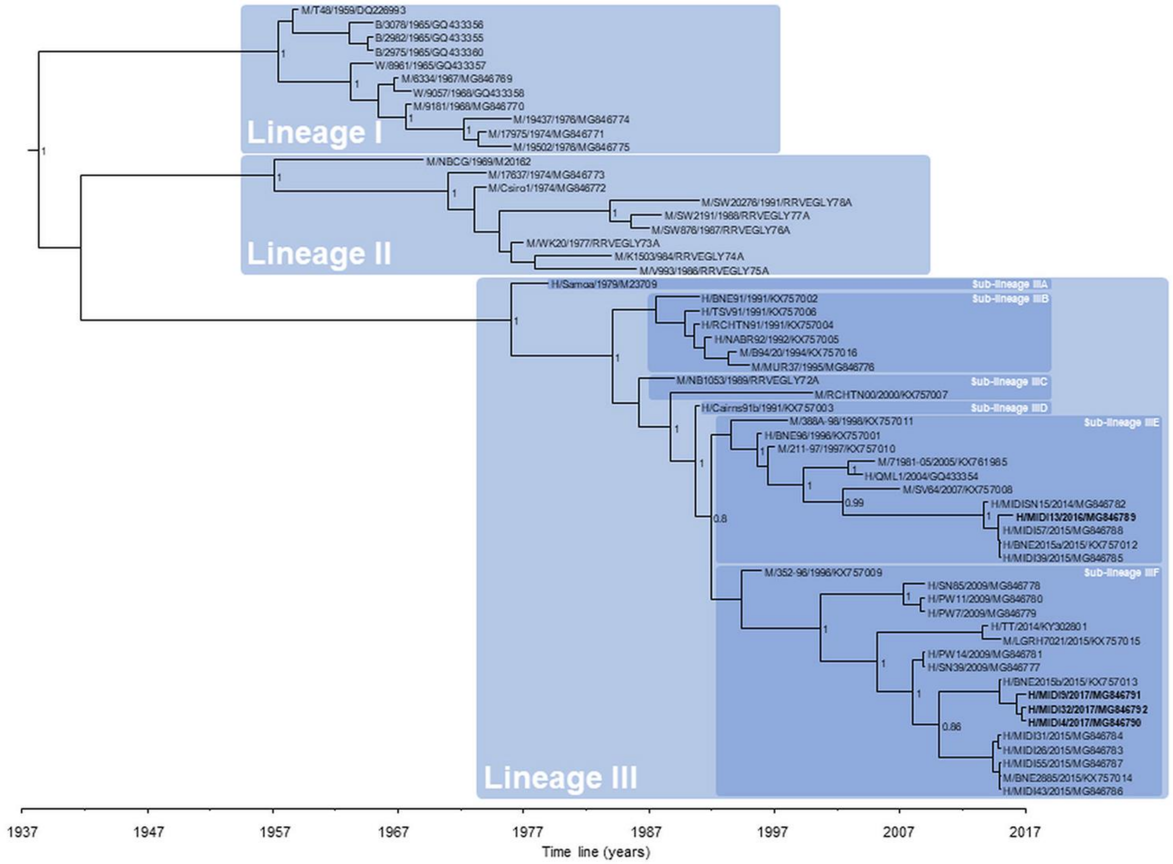

Appendix Figure 4. Maximum clade credibility tree based on analysis of 58 complete RRV E2 sequences (1266nt) classified ADF SWBTA RRV isolates (highlighted) into two distinct sub-lineages in lineage III. Bayesian phylogenetic analysis method in BEAST software package (http://beast.community) was used to analysis the aligned E2 sequences, employ the GTR+G+I substitution model with a strict clock model. Numbers at nodes indicate the posterior probability values ≥0.8. The x axis represents time in years. The naming convention of the strains were name of host/strain/year of isolation/GenBank accession number, H-human, B-birds, M-mosquitoes and W-wallabies.

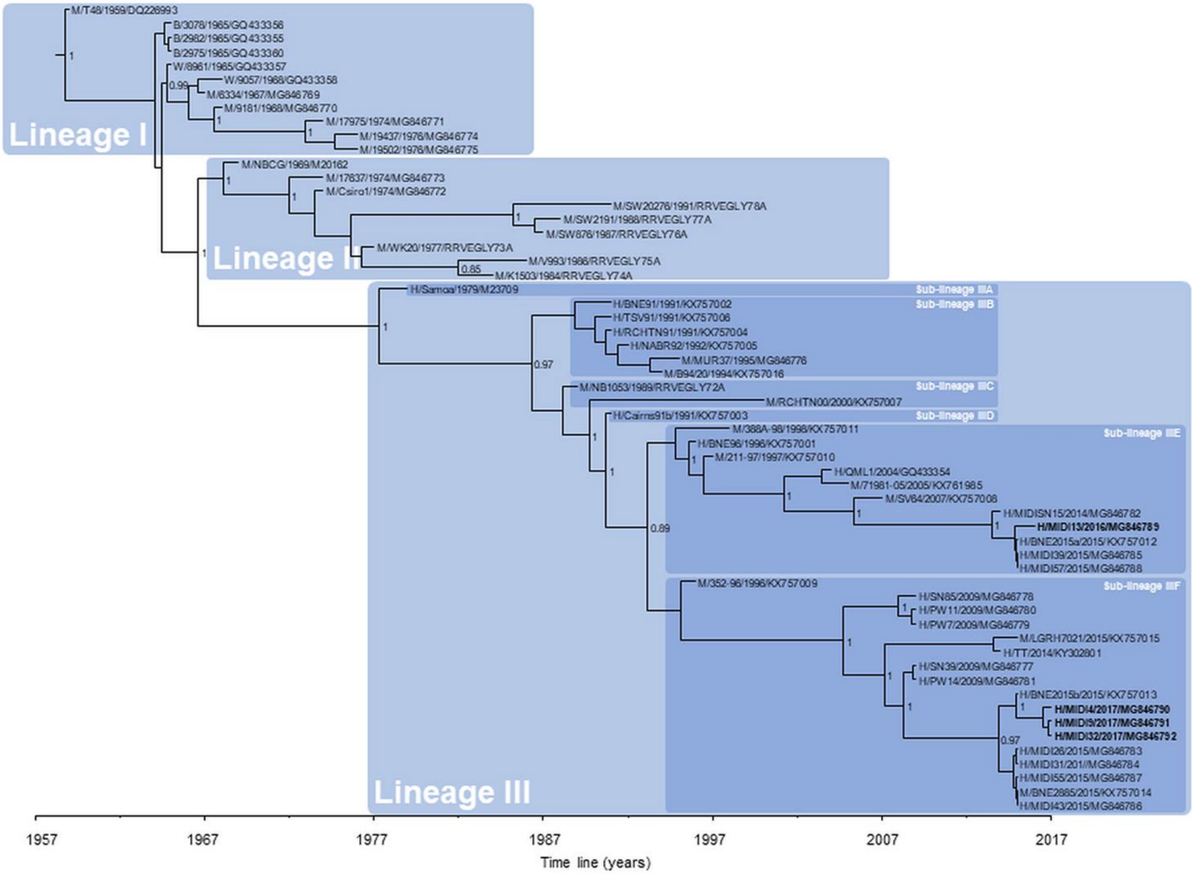

Appendix Figure 5. Maximum clade credibility tree based on analysis of 58 complete RRV E2 sequences (1266nt) classified ADF SWBTA RRV isolates (highlighted) into two distinct sub-lineages in lineage III. Bayesian phylogenetic analysis method in BEAST software package (http://beast.community) was used to analysis the aligned E2 sequences, employ the GTR+G+I substitution model with a relaxed clock mode. Numbers at nodes indicate the posterior probability values ≥0.8. The x axis represents time in years. The naming convention of the strains were name of host/strain/year of isolation/GenBank accession number, H-human, B-birds, M-mosquitoes and W-wallabies.

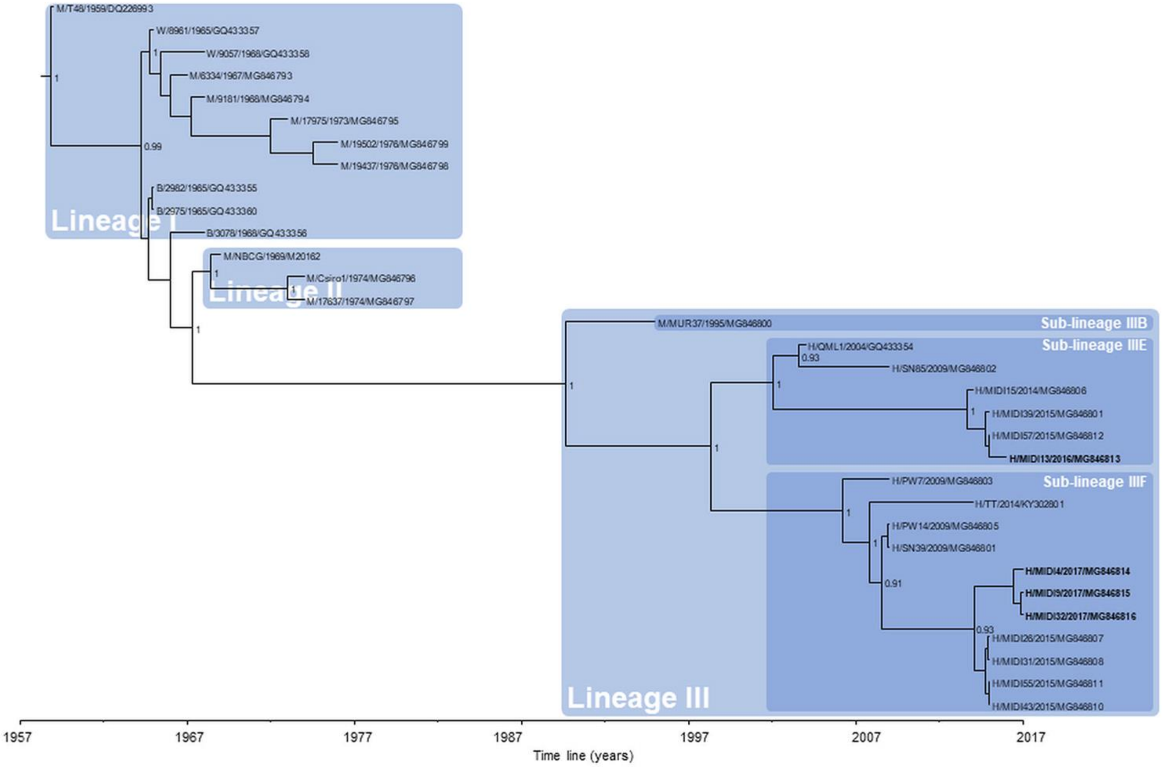

Appendix Figure 6. Maximum clade credibility tree based on analysis of 32 complete RRV nsP3 sequences (1650 nt) classified ADF SWBTA RRV isolates (highlighted) into two distinct sub-lineages in lineage III. Bayesian phylogenetic analysis method in BEAST software package (http://beast.community) was used to analysis the aligned nsP3 sequences, employ the HKY substitution model with a relaxed clock model. Numbers at nodes indicate the posterior probability values ≥0.8. The x axis represents time in years. The naming convention of the strains were name of host/strain/year of isolation/GenBank accession number, H-human, B-birds, M-mosquitoes and W-wallabies.

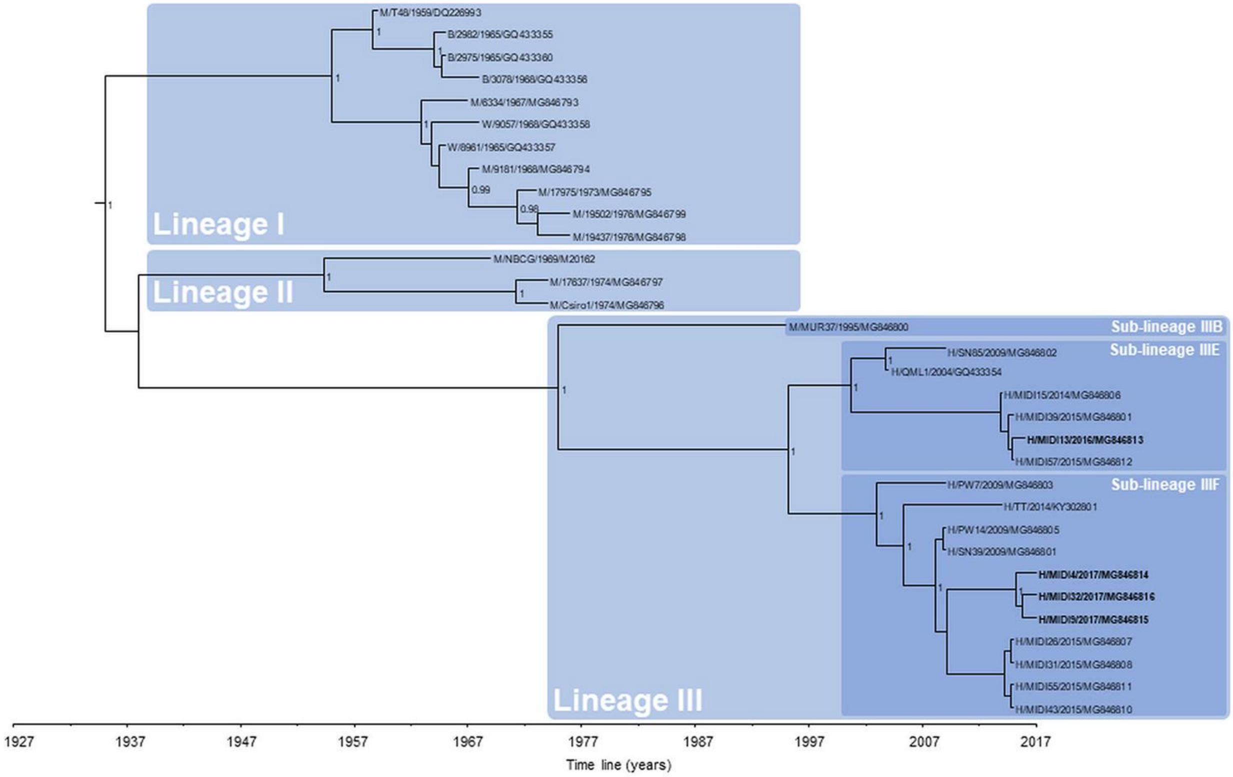

Appendix Figure 7. Maximum clade credibility tree based on analysis of 32 complete RRV nsP3 sequences (1650 nt) classified ADF SWBTA RRV isolates (highlighted) into two distinct sub-lineages in lineage III. Bayesian phylogenetic analysis method in BEAST software package (http://beast.community) was used to analysis the aligned nsP3 sequences, employ the GTR+G+I substitution model with a strict clock model. Numbers at nodes indicate the posterior probability values ≥0.8. The x axis represents time in years. The naming convention of the strains were name of host/strain/year of isolation/GenBank accession number, H-human, B-birds, M-mosquitoes and W-wallabies.

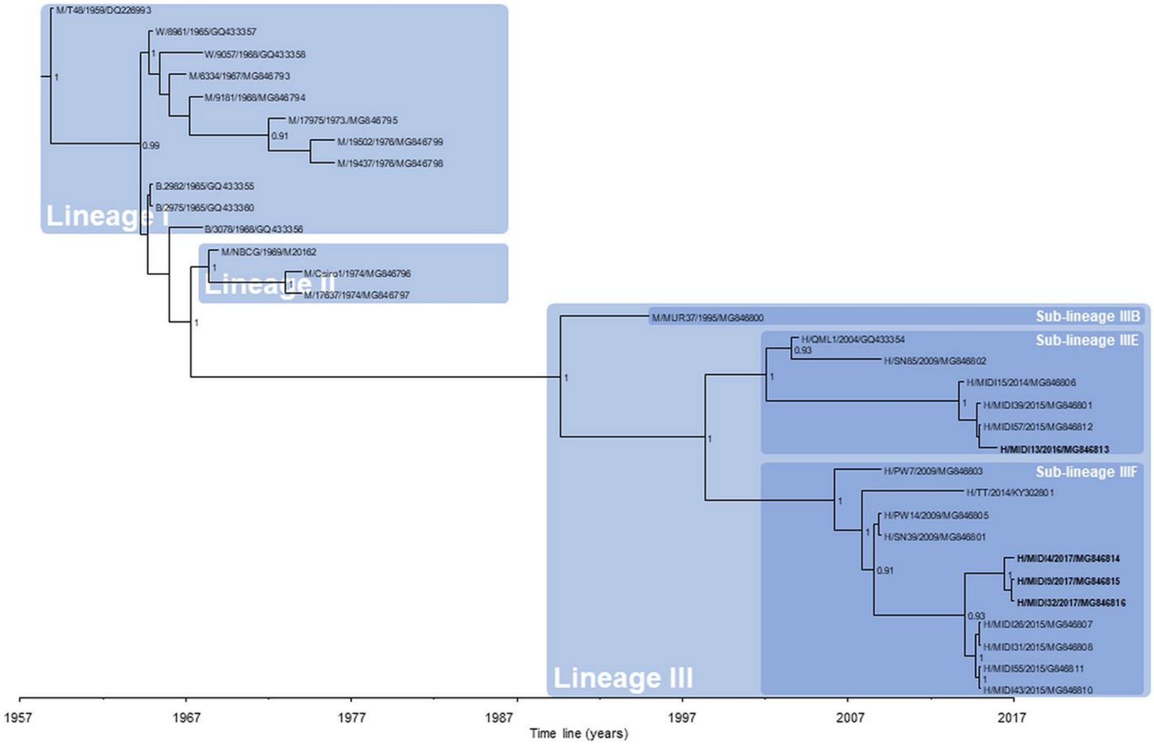

Appendix Figure 8. Maximum clade credibility tree based on analysis of 32 complete RRV nsP3 sequences (1650 nt) classified ADF SWBTA RRV isolates (highlighted) into two distinct sub-lineages in lineage III. Bayesian phylogenetic analysis method in BEAST software package (http://beast.community) was used to analysis the aligned nsP3 sequences, employ the GTR+G+I substitution model with a relaxed clock model. Numbers at nodes indicate the posterior probability values ≥0.8. The x axis represents time in years. The naming convention of the strains were name of host/strain/year of isolation/GenBank accession number, H-human, B-birds, M-mosquitoes and W-wallabies.
